# Classification related to immunogenic cell death predicts prognosis, immune microenvironment characteristics, and response to immunotherapy in lower-grade gliomas

**DOI:** 10.3389/fimmu.2023.1102094

**Published:** 2023-04-19

**Authors:** Yirui Kuang, Bincan Jiang, Hecheng Zhu, Yi Zhou, Haoxuan Huang, Can Li, Wenlong Zhang, Xuewen Li, Yudong Cao

**Affiliations:** ^1^Department of Neurosurgery, National Clinical Research Center for Geriatric Disorders, Xiangya Hospital, Central South University, Changsha, Hunan, China; ^2^Hengyang Medical School, University of South China, Hengyang, Hunan, China; ^3^Department of Oncology Radiology, Changsha Kexin Cancer Hospital, Changsha, Hunan, China

**Keywords:** lower-grade glioma, immunogenic cell death, molecular subtypes, prognostic signature, tumor immune microenvironment, immune infiltration

## Abstract

**Background:**

Immunogenic cell death (ICD) is a form of cell death that elicits immune responses against the antigens found in dead or dying tumor cells. Growing evidence implies that ICD plays a significant role in triggering antitumor immunity. The prognosis for glioma remains poor despite many biomarkers being reported, and identifying ICD-related biomarkers is imminent for better-personalized management in patients with lower-grade glioma (LGG).

**Materials and methods:**

We identified ICD-related differentially expressed genes (DEGs) by comparing gene expression profiles obtained across Genotype-Tissue Expression (GTEx) and The Cancer Genome Atlas (TCGA) cohorts. On the foundation of ICD-related DEGs, two ICD-related clusters were identified through consensus clustering. Then, survival analysis, functional enrichment analysis, somatic mutation analysis, and immune characteristics analysis were performed in the two ICD-related subtypes. Additionally, we developed and validated a risk assessment signature for LGG patients. Finally, we selected one gene (EIF2AK3) from the above risk model for experimental validation.

**Results:**

32 ICD-related DEGs were screened, dividing the LGG samples from the TCGA database into two distinct subtypes. The ICD-high subgroup showed worse overall survival (OS), greater immune infiltration, more active immune response process, and higher expression levels of HLA genes than the ICD-low subgroup. Additionally, nine ICD-related DEGs were identified to build the prognostic signature, which was highly correlated with the tumor-immune microenvironment and could unambiguously be taken as an independent prognostic factor and further verified in an external dataset. The experimental results indicated that EIF2AK3 expression was higher in tumors than paracancerous tissues, and high-expression EIF2AK3 was enriched in WHO III and IV gliomas by qPCR and IHC, and Knockdown of EIF2AK3 suppressed cell viability and mobility in glioma cells.

**Conclusion:**

We established novel ICD-related subtypes and risk signature for LGG, which may be beneficial to improving clinical outcome prediction and guiding individualized immunotherapy.

## 1 Introduction

Malignant gliomas, including lower-grade glioma (LGG, WHO II and III) and glioblastoma (GBM, WHO IV), are the most common and remain untreatable primary central nervous system (CNS) neoplasms characterized by early and rapid infiltrative growth, a high rate of postoperative recurrence, and high therapy resistance (1, 2). Maximum safe surgical resection combined with postoperative radiotherapy and chemotherapy is currently the mainstay of treatment for malignant gliomas (3). Despite advances in diagnosing and treating gliomas, there has been little impact on the outcomes of patients with malignant gliomas (4, 5). Recent studies have found that tumor immune response plays an increasingly crucial role in the development of glioma (6–9), suggesting that immunotherapy holds great promise in treating glioma. With the continued growth of immunotherapies (10), such as immune checkpoint therapy (ICT), tumor vaccines, and immunomodulators, research to forecast and recognize accurate immunotherapy biomarkers in gliomas biomarkers for immunotherapy of glioma will become more prominent.

Immunogenic cell death (ICD), a specific pattern of cell death, elicits an immune response against the corresponding antigens of dead or dying neoplastic cells (11, 12). ICD can release a range of immunostimulatory damage-associated molecular patterns (DAMPs) from dead or dying neoplastic cells, such as high mobility group protein B1 (HMGB1), extracellular ATP, endogenous nucleic acids, and cytoplasmic calreticulin (13). Immunotherapy that boosts the host immune system to fight against tumors has revolutionized cancer treatment. In recent years, a growing body of evidence has strongly suggested that ICD can initiate a host anti-cancer immune response (14). Although ICD in gliomas has been relatively evaluated in preclinical models in the past few years, the evidence that gliomas could benefit from ICD-based therapies is not suffi;ciently compelling (15). Thus, further investigation in patients with gliomas needs to conduct in clinical contexts to assess the possibility of an ICD. Remarkably, searching for novel biomarkers to sort molecular subsets in ICD immunotherapy and stratifying responders and non-responders is highly desirable.

**Figure 1:**
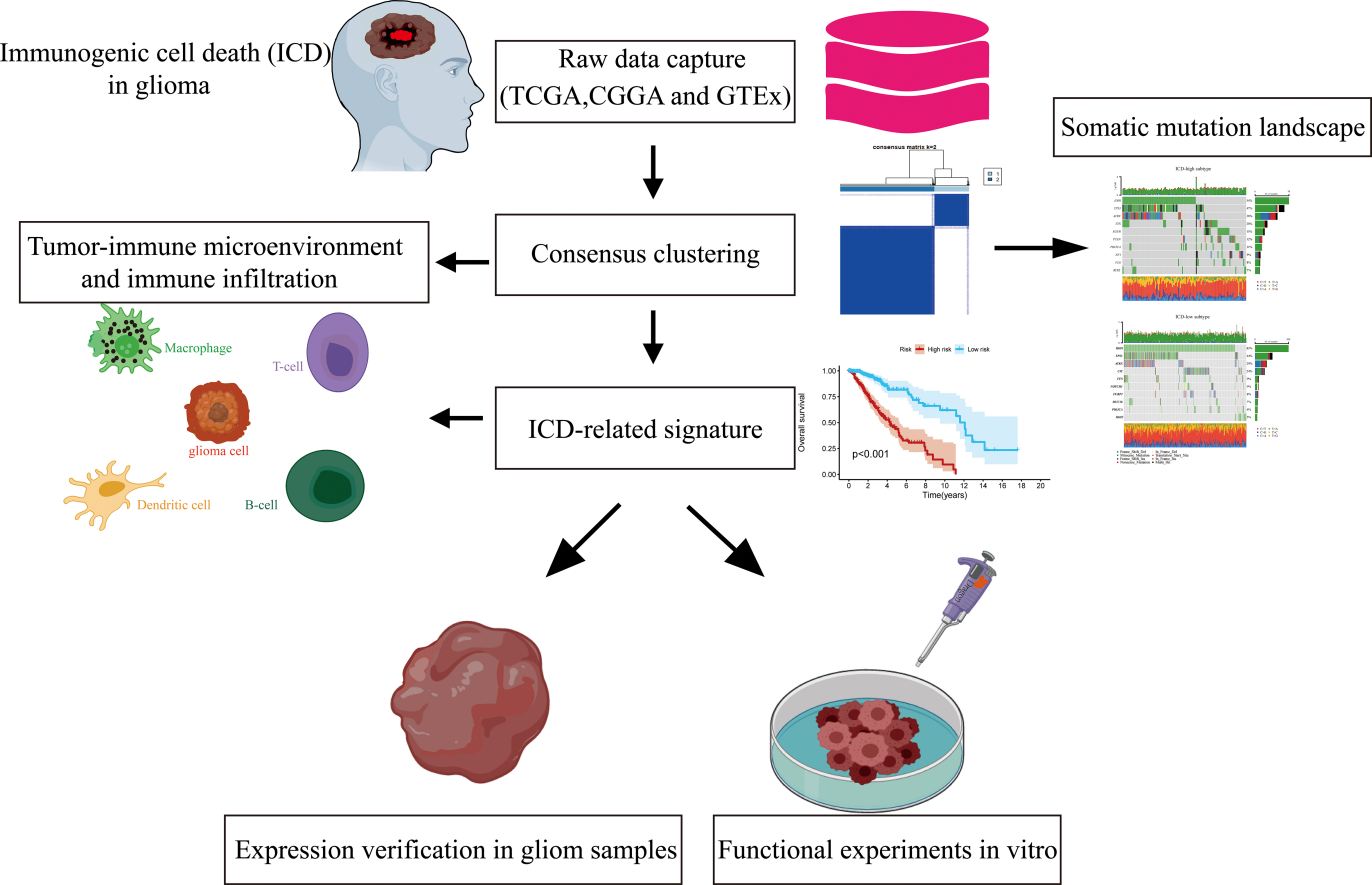
Procedures for designing and analyzing flowcharts in the present research.

We extracted LGG patients’ mRNA expression profiles and corresponding clinical characteristics from public databases in the current work. We then built two ICD-related isoforms depending on differentially expressed ICD-related genes between the LGG and normal brain tissue samples from the TCGA and GTEx cohorts. Meanwhile, an ICD-related prognostic signature for LGG was generated by univariate Cox regression and the least absolute shrinkage and selection operator (LASSO) Cox regression analyses and then validated via the CGGA cohort. The streamlined flow chart of the study methodology and design is depicted in **Figure 1**. In this study, we aim to identify relevant biomarkers as well as develop and validate a novel ICD-related subtype and risk-predictive model to assess the predictive value of tumor microenvironment and adverse prognosis and guide individualized immunotherapy in gliomas.

## 2 Materials and methods

### 2.1 Collection and processing of data from patients with LGG

Our research mainly encompassed LGG patient cohorts from the TCGA and CGGA databases as well as the normal brain tissue specimens from the GTEx database. The RNA sequencing profiles (FPKM values) from TCGA (RRID : SCR_003193)-LGG (n = 529) and GTEx (RRID : SCR_013042)-brain (n = 1152) cohorts were acquired from the UCSC Xena database (https://xenabrowser.net/) and were uniformly normalized into log2 (FPKM + 1). 509 somatic mutation data files of LGG were also analyzed from the UCSC database. The RNA sequencing data and accompanying clinical information from the CGGA dataset (DataSet ID: mRNAseq_693) were applied for external validation (16). The data about the gene set of 34 ICD-related genes were acquired from the previously published paper (**Supplementary Table 1**) (17).

### 2.2 An integrated network of ICD-related genes and screening for differentially expressed ICD-related genes

Protein-protein association networks for ICD-related genes were studied by the STRING tool (18). The Limma (RRID: SCR_010943) package (https://bioconductor.org/packages/release/bioc/html/limma.html) determined the differential expression of mRNAs. The False Discovery Rate (FDR) adjusted p-value was used to lower the chances of false-positive results. |Fold Change| > 1 and adjusted p-value < 0.05 were adopted as the differential gene screening standard.

### 2.3 Consensus clustering and survival analysis

A consensus clustering algorithm was applied to identify potential molecular subgroups of the LGG patients in the TCGA dataset in terms of differentially expressed ICD-related genes using the ConcensusClusterPlus package in R software. The K-means clustering method was employed for eight different cluster numbers K that ranged between 2 and 9 to identify the optimal number of clusters. This process was repeated 1000 times to guarantee stable results. Cluster heatmap were created in R using the pheatmap package. The Survival R package performed survival analysis to evaluate the relevance between ICD-related subtypes, risk model, and clinical outcomes.

### 2.4 Differential expression analysis between ICD-related subtypes and functional and pathway enrichment analysis

Differential expression genes in ICD-related subtypes were analyzed by the limma (RRID : SCR_010943) package (https://bioconductor.org/packages/release/bioc/html/limma.html), and the cut-off of |Fold Change| > 2 and FDR < 0.05 was applied. Volcano maps and heatmaps were conducted to visualize the differential expression genes in ICD-related subtypes using the R packages heatmap and ggplot2, respectively. Gene Ontology (GO) and Kyoto Encyclopedia of Genes and Genomes (KEGG) enrichment were analyzed using the clusterProfiler (RRID : SCR_016884) package (https://bioconductor.org/packages/release/bioc/html/clusterProfiler.html) in R to probe probable bio functions and signaling pathways between ICD-related subtypes (19). A corrected p-value (q value) < 0.05 was regarded as a remarkable enrichment for GO terms and KEGG pathways.

### 2.5 Gene set enrichment analysis

The gene set enrichment analysis (GSEA, RRID : SCR_003199) was employed to ascertain the existence of statistically significant variations in the expression of a suite of functionally related genes between ICD-related subtypes (20, 21). The clusterProfiler R package was employed to conduct the GSEA analysis, and a bunch of predefined gene sets was acquired from the Molecular Signatures Database (MSigDB) (http://software.broadinstitute.org/gsea/msigdb).

### 2.6 Evaluating the somatic mutation in ICD-related subgroups

Mutation Annotation Format (MAF) files for the somatic mutations of the LGG patients were retrieved from the TCGA official website. We employed the waterfall function in the maftools R package to visualize and summarize the mutation landscape in ICD-related subgroups from TCGA-LGG patients.

### 2.7 Evaluating the tumor immune microenvironment between the two ICD-related subgroups

The CIBERSORT deconvolution algorithm was implied to estimate 22 types of human immune cells to elucidate the immune infiltration landscape of LGG (22). The infiltration volume of 22 categories of immune cells in 509 LGG samples was calculated based on TCGA-LGG transcriptome data. The relative proportions of 22 categories of tumor-infiltrating immune cells between ICD-related subsets were compared. In the meantime, the ESTIMATE algorithm was used to calculate the immune score, tumor purity, and stromal score within the tumor microenvironment. Based on TCGA-LGG transcriptome data, we also analyzed different gene expression levels in human leukocyte antigen (HLA) family genes and immune checkpoint-related genes among ICD-related subclasses.

### 2.8 Constructing and validating the ICD-related prognostic signature

We performed the differential expression analysis of ICD-related genes between TCGA-LGG and normal brain tissue specimens from the GTEx database by applying the R package ‘limma’ as previously described and obtained 32 differentially expressed ICD-related genes. Then a univariate COX regression analysis was conducted to filter the genes significantly associated with OS (overall survival) of the TCGA-LGG cohort depending on the differentially expressed ICD-related genes. For the prognosis-related ICD-related genes, we performed LASSO regression with tenfold cross-validation using R ‘glmnet’ package in R software and screened for the optimal gene combinations for constructing the risk signature. Risk score value for each sample was computed by the following formula: $\text{Risk}\;\text{score}=\sum_{i=1}^{n}coefficient_{i}\times expression_{i}$ . On the basis of the median risk scores, patients were grouped into high-risk and low-risk subgroups in the TCGA training and CGGA validation cohorts. The survival evaluation of the ICD-related signature was completed with the KM analysis using R with the survival package.

### 2.9 Collection of tumor specimens from glioma patients

This research was approved by the Ethics Committee of Xiangya Hospital in Central South University (Ethical approval code: 202210232), and all patients subscribed to an informed consent form. No subjects were excluded from our present research. Seventy-two glioma specimens and twelve paracancerous tissues were acquired from the neurosurgery department at Xiangya Hospital in Central South University from January 2021 to August 2022. There were 45 male and 27 female cases, aged 11 to 74 years old (median 52 years), including 19 cases of WHO grade II, 15 cases of WHO grade III, and 38 cases of WHO grade IV. The patients did not receive any radio- or chemotherapy before surgery. Subjects were not randomly grouped as this was not considered relevant to this study, and we did not use a power analysis to examine sample size because our research did not report statistics between groups or within group variables. All tumor specimens and seven pairs of cancer and paracancerous specimens were analyzed by real-time PCR (RT-qPCR). In addition, seventy glioma samples and twelve paraneoplastic samples were subjected to immunohistochemical analysis.

### 2.10 RNA extraction and real-time PCR

We extracted total RNA from newly available glioma samples, corresponding paracancerous tissues, and cultured glioma cells using TRIzol (Invitrogen Life Technologies). Complementary DNA (cDNA) was synthesized from 1 μg total RNA using the Reverse Transcription Kit (Thermo Fisher Scientific). Real-time PCR (RT-qPCR) was executed using Taq Pro Universal SYBR qPCR Master Mix (Vazyme). Expression of the selected gene was quantified relative to TUBB using the 2–ΔΔCt method. qPCR primer sequences applied in this work are listed below: EIF2AK3 forward primer 5-ACGATGAGACAGAGTTGCGAC-3, EIF2AK3 reverse primer 5-ATCCAAGGCAGCAATTCTCCC-3; TUBB forward primer 5-TGGACTCTGTTCGCTCAGGT-3, TUBB reverse primer 5-TGCCTCCTTCCGTACCACAT-3.

### 2.11 Tissue microarray, immunohistochemistry, and scoring

As previously described (23), the tissue microarray, including 70 samples in distinct grade-level gliomas and 12 paracancerous tissues, was constructed and applied to probe the expression of EIF2AK3.

We followed a protocol previously published for immunohistochemistry (IHC) (24). After routine dewaxed and hydrating treatment, the glioma chip was exposed to antigenic heat repair in citrate buffer (10 mM citric acid and pH 6.0). The endogenous peroxidase was then blocked using 3% H2O2-methanol. To block non-specific sites, goat serum that is not immune was employed. After that, the slide was first incubated with the primary antibody against EIF2AK3 (rabbit, 1:200, Proteintech Cat# 20582-1-AP, RRID : AB_10695760) at 4°C for an overnight period, followed by 2 hours at room temperature with a secondary antibody in the dark. Then, the chip was washed and treated with 3, 3’- diamino-benzedine (DAB) for around 5 minutes. Hematoxylin was utilized to counterstain the segment. Finally, the chip was flushed, dehydrated, mounted with a coverslip, and examined under a microscope. As a negative control, PBS was utilized instead of the primary EIF2AK3 antibody under the same trial conditions.

### 2.12 Cell-line culture and transfection

The LGG cell lines, including SHG44 (RRID : CVCL_6728) and HS683 (RRID : CVCL_0844), and normal human astrocyte cell line (HEB) were acquired from Xiangya Medical College (Central South University, Changsha, Hunan, China) and were grown under standard conditions in high glucose DMEM medium (Gibco; Thermo Fisher Scientific) incorporating 10% fetal bovine serum (QmSuero, Wuhan, China). siRNAs were transfected into glioma cells via liposome transfection (Lipofectamine 2000; Invitrogen) for 48h prior to functional cell experiments. RNA siRNAs were programmed and manufactured by Guangzhou RiboBio (RiboBio, Guangzhou, China); We evaluated three siRNA sequences to find the most effective one.

### 2.13 Cell viability and wound healing assays

Cell viability was detected with Cell Counting Kit-8 (CCK8; NCM Biotech, Suzhou, China) according to the manufacturer’s instructions. Briefly, 1×103 cells per well in 100 µL of complete medium were seeded into a 96-well plate. After cultured for different time points, 100 µL CCK8 solution was added into each well (CCK8: medium = 1:10) after the supernatants were removed and incubated for one and a half hours before analysis. Cell proliferation ability was examined by measurement of the optical density at 450 nm.

We evaluated SHG44/HS683 cell mobility by using wound healing assays. After 36 hours of transfection, a confluent monolayer of glioma cells was formed by seeding each group at a density of 5 × 105 cells/well in a 6-well culture plate. Using a sterile 200 µl pipette tip, monolayers were scratch wounded. The cells were in fresh high glucose DMEM medium with serum-free for 24 hours at 37°C. Under a phase-contrast microscope, scratch wounds were observed, and images were captured at 0 and 24 hours. Three replicates were performed for each assay.

### 2.14 Statistical analysis

All statistical analyses and graphs were performed using the R software (version 4.1.2, RRID : SCR_001905) and GraphPad Prism (version 7.0.0, RRID : SCR_002798). Log-rank tests were applied for Kaplan-Meier survival analysis. The Student’s t-test or Mann-Whitney U-test was employed to compare two groups. Kruskal-Wallis tests were employed to compare multiple groups. The statistical correlation was measured using Spearman’s or Pearson’s tests. The discrepancy at P less than 0.05 was deemed meaningful.

## 3 Results

### 3.1 Screening differentially expressed ICD-related genes and identification of two ICD-related subgroups in LGG patients from the TCGA cohort

**Figure 2:**
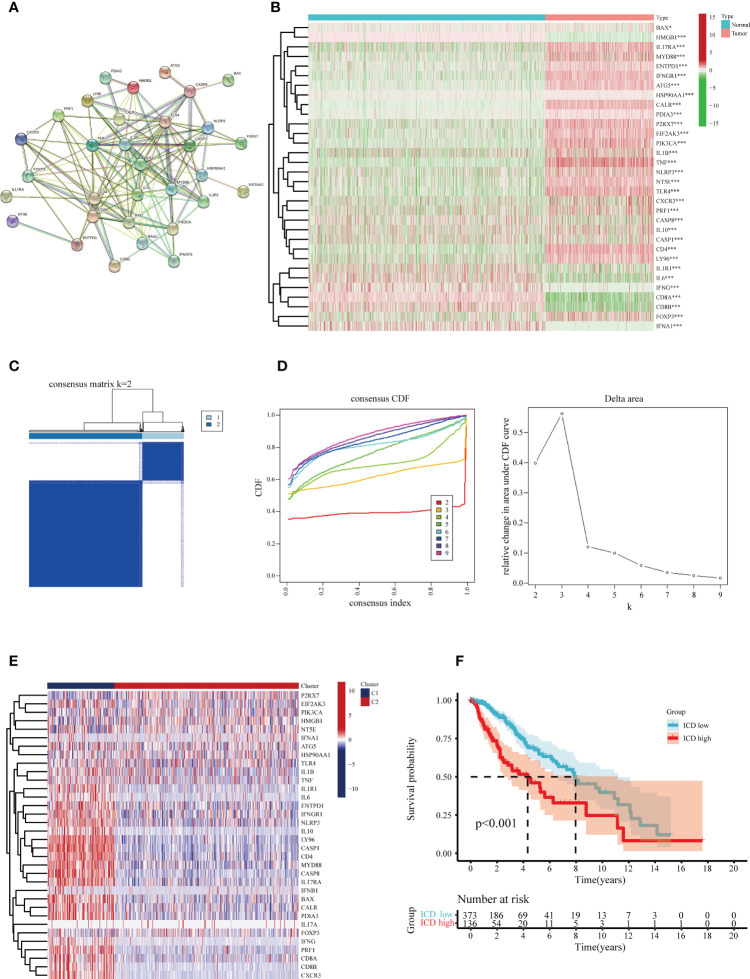
Identification of the differentially expressed ICD-related genes and sub-clusters derived from these genes. **(A)** Protein-protein interactions (PPI) among the differentially expressed ICD-related genes. **(B)** Heatmap visualization of expression levels in 32 differentially expressed ICD-associated genes between lower-grade glioma (LGG) samples and normal brain tissue specimens, respectively, from the TCGA and GTEx databases. **(C)** In accordance with the consensus clustering matrix (k =2), LGG patients were split into two sub-clusters. **(D)** Consensus clustering cumulative distribution function (CDF) with k=2-10. **(E)** Heatmap depicts the expression levels of 34 ICD- associated genes between various subclasses. The blue color denotes low expression, and the red means high expression. **(F)** Kaplan-Meier (KM) curves for patients’ overall survival (OS) between the two clusters. *P < 0.05; ***P < 0.001.

Following a systematic literature survey, Abhishek et al. (17) have previously summarized ICD-associated genes. To further reveal associations between these ICD-related genes, we subjected the candidates to protein-protein interaction (PPI) network analysis via the STRING database (**Figure 2A**). Our next step was to characterize gene expression in ICD between glioma specimens and normal brain tissue. In glioma, the majority of ICD-associated genes were up-regulated, including BAX, IL17RA, MYD88, ENTPD1, IFNGR1, ATG5, CALR, P2RX7, EIF2AK3, PIK3CA, IL1B, TNF, NLRP3, NT5E, TLR4, CD4, LY96, and FOXP3 (**Figure 2B**).

The consensus clustering analysis was performed to investigate the associations between the expression of the ICD-related genes and glioma samples. The letter k represented the number of clusters. We found the highest intra-group collinearity and lower inter-group collinearity when k=2. The glioma samples in the TCGA cohort were segregated into two categories on account of the distinct expression patterns of ICD-related genes via k-means clustering (**Figures 2C, D**). Overall, higher expression of the ICD-related genes in cluster 1 indicates an ICD-high subtype. In contrast, ICD-related genes in cluster 2 were downregulated, indicating an ICD-low subtype (**Figure 2E**). In addition, survival analysis revealed that the ICD-low subset manifested a more extended survival period than the high ICD group, with a notable discrepancy (**Figure 2F**).

### 3.2 Screening for differentially expressed genes in two distinguishable ICD-related subsets and determining their biological functions

**Figure 3:**
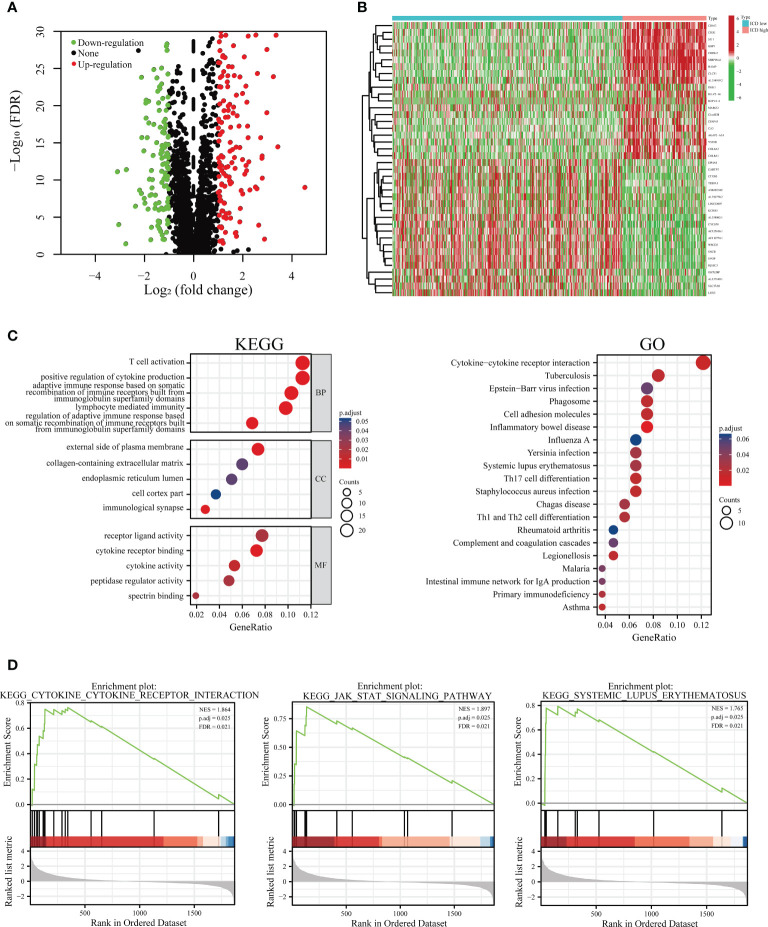
Functional and pathway enrichment identification of genes differentially expressed between ICD-high and ICD-low subsets. **(A)** Based on the TCGA cohort, a volcano plot demonstrated differentially expressed genes (DEGs) in distinct ICD-associated subsets. **(B)** A heatmap shows the top 20 up-regulated and down-regulated DEGs in the two subtypes. **(C)** Enrichment analyses of GO terms and KEGG pipelines for DEGs. **(D)** Gene set enrichment analysis (GSEA) was performed further to screen the significant pathway between ICD-high and ICD-low subtypes.

To address the characteristics of the ICD-low subset with a promising prognosis compared to the ICD-high subset, we determined the critical DEGs and corresponding signaling pipelines within each subpopulation to uncover the underlying molecular and cellular mechanisms that regulate their clinical outcomes. Altogether, we found 307 dysregulated genes (**Figure 3A**; **Supplementary Table 2**), and the heat map presented the top 20 up-regulated and 20 down-regulated genes that are differentially expressed between ICD-low and ICD-high subgroups (**Figure 3B**). The KEGG enrichment analysis illustrated that the up-regulated genes in the ICD-high subtype were involved in T cell activation, receptor-ligand activity, positive regulation of cytokine production, and the external side of plasma membrane. GO functional enrichment analyses displayed the DEGs were chiefly involved in cytokine-cytokine receptor interaction, Th1 and Th2 cell differentiation, and Th17 cell differentiation (**Figure 3C**). Our result revealed the ICD-associated subsets were linked to the immunological microenvironment, especially in the ICD-high subset.

Additionally, to step up to explore the relevant signaling circuits involved in activation in the ICD-high subset, we conducted the GSEA via a comparison between the ICD-high and ICD-low subsets. GSEA results suggested that up-regulated pathways were fundamentally linked to immune system-related processes, such as JAK-STAT signaling pathway, cytokine-cytokine receptor interaction, and systemic lupus erythematosus pathway (**Figures 3D**).

### 3.3 The landscape of somatic mutations and tumor immune microenvironment in two ICD-related subtypes

**Figure 4:**
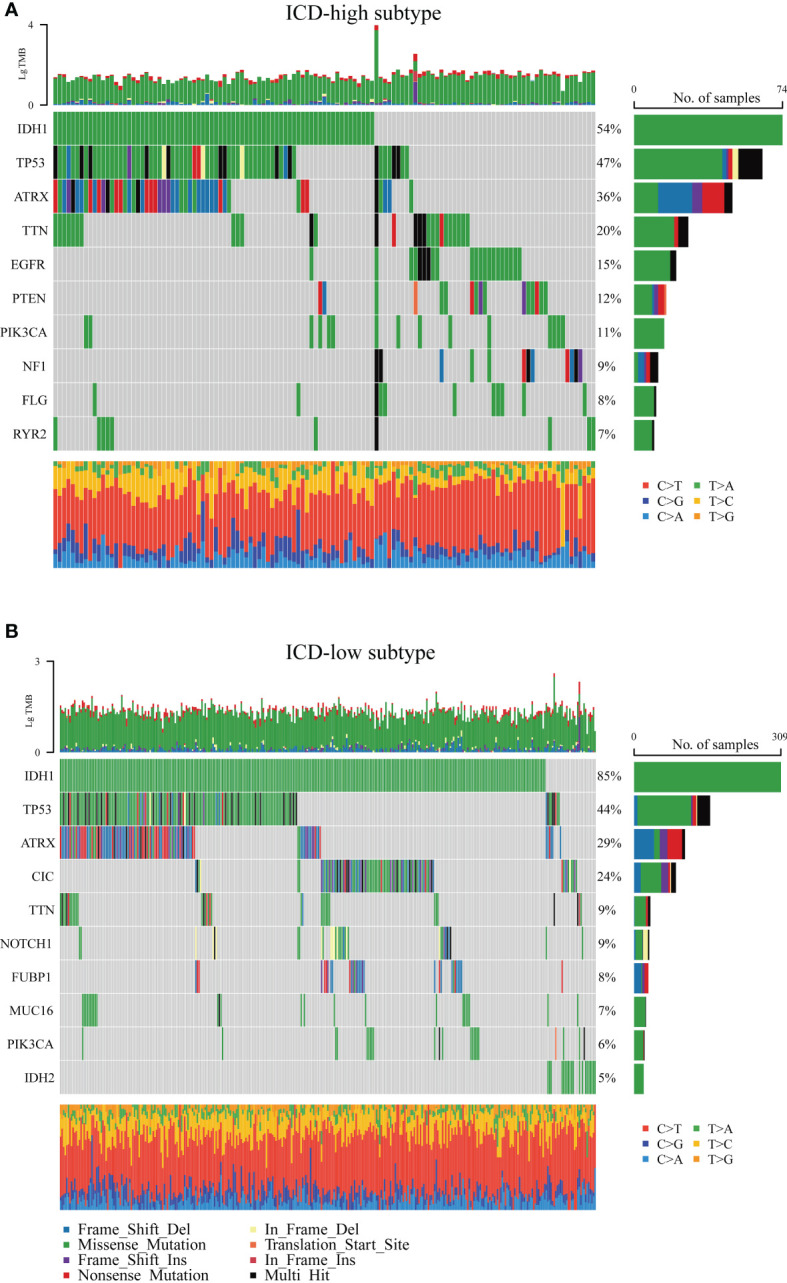
Comparison of the top 10 most frequently mutated genes of two ICD subtypes. **(A)** ICD-high subtype. **(B)** ICD-low subtype.

Next, the somatic mutation data of patients with LGG acquired from the TCGA database were processed with VarScan software. We found clear somatic mutation profiles between the two subsets, and the gene mutation patterns between the two subgroups were not identical (**Figures 4A, B**). The most frequent mutated genes common to both subgroups were IDH1, TP53, and ATRX; missense mutation was the most common aberration. However, the relative frequencies and several mutated genes of transcriptomic subtypes were discovered to varied. IDH1 (54%), TP53 (47%), ATRX (36%), and TTN (20%) occupied the top four positions with the greatest possible mutation frequencies in the ICD-high group, and IDH1 (85%), TP53 (44%), ATRX (29%), and CIC (24%) in the ICD-low group, while IDH1 was fairly high mutated in the ICD-low group. Interestingly, although the CIC, NOTCH1, FUBP1, MUC16, and IDH2 genes were frequently mutated in ICD-low subtypes, there is a distinct lack of mutations in ICD-high subtypes.

**Figure 5:**
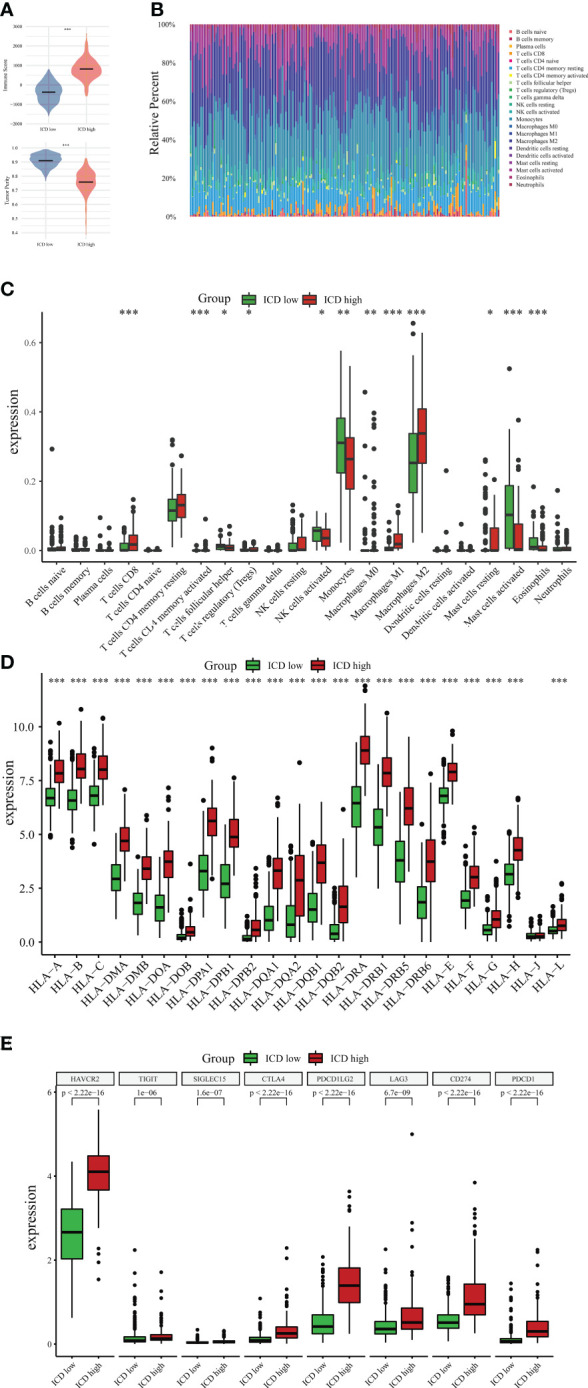
Immune landscape between ICD-high and ICD-low subtypes. **(A)** The violin plots specifically showed the differences between the two subtypes regarding the immune and tumor purity scores. **(B)** Comparative proportions of immune cell infiltration of immune cells between two ICD-associated subtypes. Box plots present different immune cells **(C)** and differential expression of HLA genes **(D)** as well as multiple immune checkpoints **(E)** between ICD-low and ICD-high subsets. *P < 0.05; **P < 0.01; ***P < 0.001.

There is increasing evidence that ICD can strengthen adaptable immune responses that are targeted against certain remaining neoplastic cells and tissues. Next, our research analyzed the differences between the two subtypes in the tumor microenvironment. Noticeably, compared to the ICD-low subgroup, the ICD-high subgroup had a higher immune score and a lower degree of tumor purity (**Figure 5A**). CIBERSORT algorithm with LM22, a signature matrix that distinguishes 22 immune cell subtypes, was employed to determine the immune cell fraction between the two ICD-related subsets (**Figure 5B**). Patients in the ICD-high subset exhibited greater percentages of activated CD4-positive Memory T-cells, CD8-positive T-cells, M1/M2 macrophages, regulatory T-cells, and resting mast cells (**Figure 5C**). Human leukocyte antigen (HLA) is believed to be the most widely distributed molecule and is widely polymorphic. HLA is primarily responsible for initiating cellular immune responses. An increase in HLA genes was observed in the ICD-high subtype (**Figure 5D**). Furthermore, immune checkpoints, such as HAVCR2, CTLA4, PDCD1LG2, LAG3, CD274, and PDCD1, were also upregulated in the ICD-high group (**Figure 5E**). In the ICD-low subgroup, the tendency appears to be somewhat in the other direction. These data might indicate that the ICD-high subset was relevant to the immunological state of hot tumors, and the ICD-low subset was connected to the immune status of cool tumors.

### 3.4 Development and validation of an ICD-related prognostic signature

**Figure 6:**
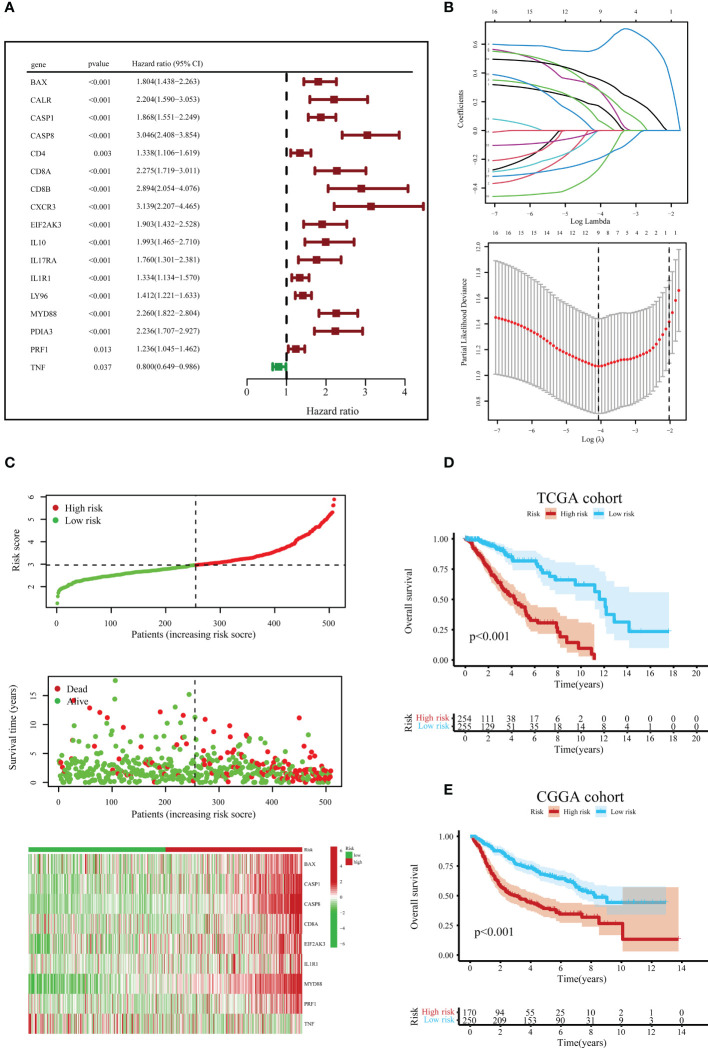
Construction and verification of the ICD risk signature. **(A)** Univariate Cox regression models identified 17 ICD-related genes associated with overall survival (OS). **(B)** Through LASSO Cox regression analysis, nine prognostic ICD-related genes were identified in the TCGA queue. **(C)** Distribution map of the risk score, individual case survival information, and a clustering heatmap of mRNA expression profiles of the nine signature genes in the TCGA cohort. **(D)** Kaplan-Meier (KM) curves for the OS of LGG patients in the low- and high-risk subclasses in the TCGA queue. **(E)** Likewise, the KM curves for OS in the CGGA queue.

Before constructing the ICD-related gene prognostic model, univariate Cox regression analysis was employed to locate ICD-correlated genes that were initially associated with survival, and 17 ICD-associated genes were correlated with the prognosis of LGG patients (**Figure 6A**). The LASSO Cox regression analysis screened nine ICD-linked genes according to the optimized λ score and structured them as a predictive risk model (**Figure 6B**). The risk scores in the above ICD-related model were computed as follows: Risk scores = (0.1732)*BAX + (0.0724)*CASP1 + (0.5688)*CASP8 + (0.2872)*CD8A + (0.2824)*EIF2AK3 + (-0.0008)*IL1R1 + (0.3583)*MYD88 + (-0.2717)*PRF1 + (-0.1969)*TNF.

According to the median value of risk scores in the TCGA cohort as the cut-off value, patients with LGG in the TCGA and CGGA queues were classified into low- and high-risk subgroups, respectively. Next, we surveyed the correlation between risk score distribution and survival status. In the TCGA cohort, the mortality risk of LGG patients increased, and the likelihood of their survival decreased when risk scores rose (**Figure 6C**). Further, the KM survival analysis demonstrated that in the TCGA cohort, LGG patients in the high-risk subgroup had reduced survival times or lower chances of survival versus the low-risk subgroup (**Figure 6D**). Similar results were obtained to further corroborate from the CGGA cohort (**Figure 6E**).

### 3.5 Tumor microenvironment analysis and independent prognostic value of the signature

**Figure 7:**
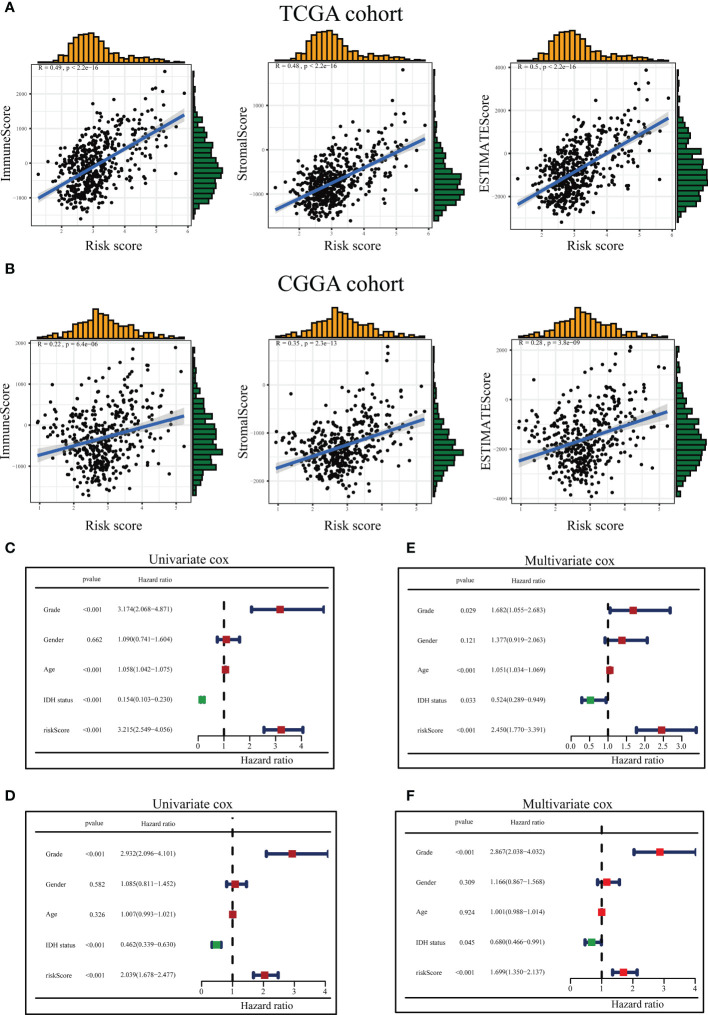
Association between the tumor microenvironment and ICD risk signature and evaluation of risk scores and predictive power of clinical variables. The relationship between the risk score and immune score, stromal score as well as ESTIMATE score in the TCGA queue **(A)** and the CGGA queue **(B)**. Forest diagram of Univariate analysis of the risk signature scores combined with well-known clinical risk variables for the TCGA-LGG queue **(C)** and the CGGA-LGG queue **(D)**. Forest diagram of Multivariate analysis of the risk signature scores combined with well-known clinical risk variables for the TCGA-LGG queue **(E)** and the CGGA-LGG queue **(F)**.

The ICD-related risk score model and the tumor immune microenvironment scores were analyzed for relevance in the tumor immune microenvironment. The results revealed that the tumor immune microenvironment scores were in positive correlation with ICD-related risk scores in both the TCGA and CGGA queues (**Figures 7A, B**).

On the TCGA and CGGA queues, univariate and multivariable Cox regression analyses were conducted to assess whether the ICD-related risky predictive model could be recognized as a stand-alone prognostic variable. The univariate Cox regression analysis presented the risk scores, grade, age, and IDH1 status could be assumed to be prognostic variables in the TCGA queue (**Figure 7C**), and risk scores, grade, and IDH1 status could be regarded as prognostic variables in the CGGA queue (**Figure 7D**). Multivariate Cox regression analysis disclosed the risk scores, neoplasm grade, age, and IDH1 status could be served as stand-alone prognostic elements for the TCGA team (**Figure 7E**), and risk scores, neoplasm grade, and IDH1 status could be treated as stand-alone prophetic elements for the CGGA team (**Figure 7F**).

### 3.6 Verification of EIF2AK3 expression glioma samples

**Figure 8:**
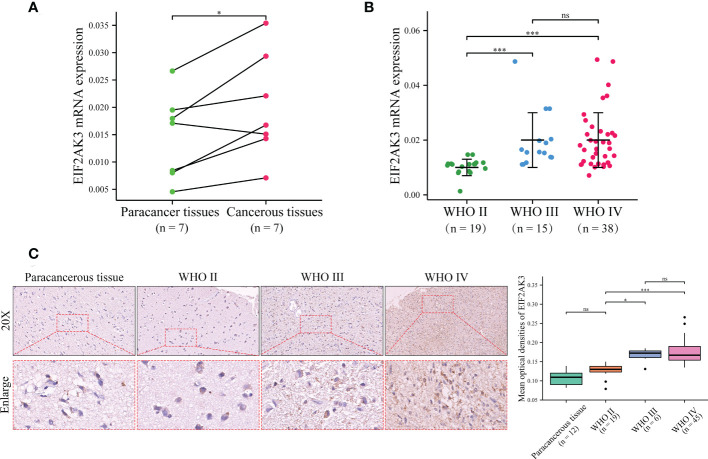
Validation of the EIF2AK3 expression in glioma tissues and non-tumor tissue analyzed through RT-qPCR and IHC. **(A)** The mRNA expression level of EIF2AK3 in diffuse glioma with glioma and para-carcinoma tissues via RT-qPCR. **(B)** The mRNA expression level of EIF2AK3 in different grades of glioma samples via RT-qPCR. **(C)** The protein expression level of EIF2AK3 in diffuse glioma specimens with different grades has been analyzed via IHC staining. ns, P > 0.05; *P < 0.05; ***P < 0.001.

To validate the expression of EIF2AK3 in glioma samples, we detected a series of glioma samples from Xiangya Hospital. We first measured the mRNA expression level of EIF2AK3 in glioma tissues and corresponding para-carcinoma tissues in seven patients via RT-qPCR. As depicted in **Figure 8A**, EIF2AK3 mRNA expression levels were significantly higher in glioma tissues than in tissues adjacent to carcinomas (P < 0.05). Then, we detected EIF2AK3 mRNA expression in 72 glioma samples of different grades. The results showed that WHO IV and WHO III gliomas had significantly higher EIF2AK3 mRNA expression than WHO II (**Figure 8B**). Next, we used tissue microarrays containing 12 cancerous and 70 diffuse glioma samples to measure EIF2AK3 expression in gliomas via IHC staining. We found the expression of EIF2AK3 protein was significantly higher in WHO grades III (P < 0.05) and IV gliomas (P < 0.001) compared to WHO II gliomas (P < 0.05); However, the difference in EIF2AK3 protein expression between WHO II glioma and para-carcinoma tissues were not significant (**Figure 8C**).

### 3.7 EIF2AK3 is engaged in the proliferation and migration of glioma cells

**Figure 9:**
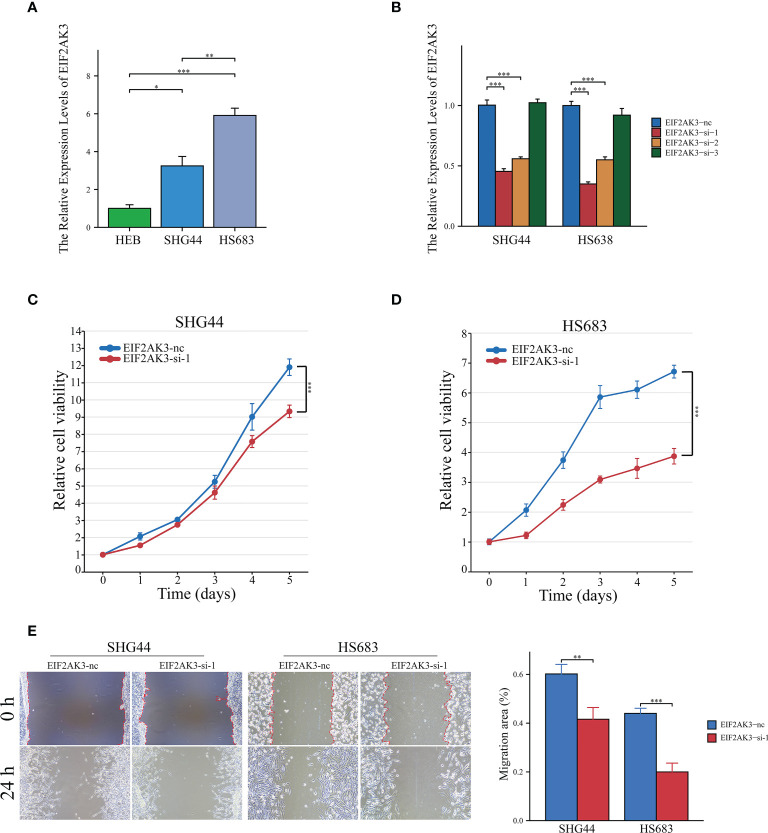
Interference of EIF2AK3 inhibits viability and mobility of glioma cells. **(A)** RT-qPCR presented the relative expression of EIF2AK3 in HEB, SHG44, and HS683 cell lines. **(B)** RT-qPCR analysis was conducted to evaluate the interference efficiency of EIF2AK3-specific short interfering RNAs (siRNAs) in SHG44 and HS638 cells; EIF2AK3-si-1 was the most effective interfering sequence and thus applied for subsequent functional experiments. **(C, D)** The effects of EIF2AK3 knockdown on the proliferation of SHG44 and HS683 cells were investigated via CCK8 assays. **(E)** Scratch experiments revealed that EIF2AK3 knockdown inhibited migration ability in SHG44 and HS683 cell lines. *P < 0.05; **P < 0.01; ***P < 0.001.

EIF2AK3 expression levels were measured in LGG cell lines (SHG44 and HS683) and normal human astrocytes (HEB), and the results showed that EIF2AK3 expression was significantly higher in the LGG cell lines compared to the normal astrocyte HEB cell line (**Figure 9A**). **Figure 9B** indicated that EIF2AK3 expression was effectively and stably downregulated via RNA interference. Since EIF2AK3-si-1 was the most effective siRNA sequence (**Figure 9B**), subsequent functional experiments were conducted with EIF2AK3-si-1. The growth curves acquired from the CCK8 proliferation assay presented that EIF2AK3 knockdown dramatically inhibited cell proliferation in SHG44 and HS683 cell lines (**Figures 9C, D**). Likewise, the wound healing assay showed that EIF2AK3 knockdown significantly hampered the migration ability of SHG44 and HS683 cell lines in comparison to the negative control group **(** **Figure 9E**).

## 4 Discussion

ICD, a type of regulated cell death (RCD), is adequate to trigger an adapted immune response in an immunologically active environment (14, 25). ICD is spreading rapidly in the area of fighting cancer treatment. It also has been shown that some anti-cancer therapies can lead to some form of immunogenic cell death in gliomas (15, 26). These procedures are implicated in changes in gene expression, including modifications in ICD-related genes. Although IDH1 mutation, 1p/19q co-deletion status (27), and several other predictive signatures associated with inflammation (28), ferroptosis, cuproptosis, necroptosis, and N6-methyladenine methylation (29) have been identified in gliomas, these ingredients are not enough to overpower the quandary of treatment and prognosis in glioma. It remains unclear, however, whether ICD-based genomic biomarkers are related to clinical outcomes in gliomas. Consequently, identifying ICD-related biomarkers for distinguishing glioma patients could be advantageous.

Our study found that the differentially expressed ICD-related genes can categorize LGG patients into two subclasses, which manifested remarkable variations in potential mechanisms, somatic mutations, and immunity. ICD-high subclass was linked to unfavorable clinical outcomes and immune-hot phenotype. Besides, A risky predictive signature integrating nine ICD-related genes was established in the TCGA queue and verified in the CGGA queue, segregating the glioma patients into low- and high-risk subgroups. This risk prediction model presented a high-level forecasting ability for short-term survival as well as tumor microenvironment and might act as an indicator of independent prognosis for glioma patients.

Over the past few decades, the immuno-oncological microenvironment has gradually become a hot spot in cancer research (30). The tumor microenvironment is the central battlefield for tumor cell-immune cell interactions (31). The lack of efficacy of immunotherapy in the clinical management of patients with glioma is partially explained by the immunosuppressive traits triggered by glioma-infiltrating immune cells (32). The immuno-inhibitory microenvironment is a leading contributor to immunotherapeutic tolerance in glioma (33). Glioma therapy induces ICD, which remodels the tumor immune microenvironment (34, 35) and activates the immune system against carcinoma in immunocompetent patients. Therefore, we identified two ICD subclasses of patients through consensus clustering. Our finding that the ICD-high subclass was correlated to more active tumor immune status compared to the ICD-low subclass is congruent with previous studies. However, the ICD-high subclass exhibited a poor prognosis. Neoplastic cells may escape ICDs by modulating long-term dysregulation of cellular proteostasis processes, such as the PERK-eiF2α axis leading to the retention of DAMPs, thus compromising the skilled relationship between the immune system and dying carcinoma cells (15, 36). Immune cells in the neoplastic microenvironment impact the process of neoplasm progressivity. For instance, the granulocyte colony-stimulating factor secreted by mutated IDH1 glioma could enhance the efficacy of immunotherapy for patients with LGG by reprogramming the tumor microenvironment and promoting the generation of non-immunosuppressive myeloid cells (32). But, there is still no understanding of how immune signaling affects ICD-associated gene expression in gliomas (37). These observations need to be further elucidated.

In our study, we examined ICD-related genes already summarized by Abhishek et al. The present study constructed the prognostic signature, and nine ICD-related genes (BAX, CASP1, CASP8, CD8A, EIF2AK3, IL1R1, MYD88, PRF1, and TNF) were ascertained for incorporation into the risk score model for OS. The current research found a significant increase in Caspase-1 expression in glioma tissue and cell lines, mediating pyroptosis (an inflammatory form of cell death) (38). Caspase-1 had an essential position in glioma growth, mobility, aggression, epithelial-mesenchymal transition, and anti-apoptosis (39). Caspase-8 is a crucial player in extrinsic apoptosis, with downregulated activity in cancer. However, recent studies reported that Caspase-8 expression was retained, and high-level expression of Caspase-8 may be associated with a worsening prognosis in glioblastomas (40, 41). The CD8 antigen is a cell surface glycoprotein discovered on the majority of cytotoxic T lymphocytes and is responsible for mediating effective cell-cell interactions within the immune system. T lymphocyte infiltration has been detected in gliomas. Nevertheless, in certain circumstances, GBM may experience immune flight through not rendering neoplastic antigens or MHC-1, thereby blocking recognition by CD8-positive T-cells (42, 43). EIF2AK3, best known as PERK, was reported to promote glioma cell viability, migration, and anti-apoptosis *in vitro* (44, 45). Our *in vitro* experiments also confirmed that the expression level of EIF2AK3 elevated with an increase in tumor grade, and EIF2AK3 enhances the abilities of tumor cell proliferation and migration.

A previous study showed that higher expression levels of IL1R1 proteins were detected in GBM samples than in normal specimens by immunohistochemical experiments, which may have a meaningful impact on the prognosis of neoplasms (46). MYD88 expression was closely tied to the OS and WHO classification of glioma patients, participating in the virulent loop of neoplastic cell evolution and M2 macrophage polarization (47). PRF1, which served as a definite marker of the killing ability of immune cells, is associated with better survival in multiple cancers, such as bladder cancer, melanoma, and head and neck squamous cell carcinoma (48, 49). Tumor necrosis factor (TNF) matters in immune regulation and controlling tumor growth (50). Nakagawa J et al. discovered that TNF expressed by cancer-relevant macrophages could clear glioma (51). The above finding implied that the ICD-associated risk predictive model for OS could independently forecast the clinical outcomes of glioma patients and had a promising prospect for application based on the validation set.

## 5 Conclusion

In summary, we structured the ICD-relational signature for OS to independently anticipate the prognosis of LGG patients and deliver potential treatment targets. Our study accentuates the link between ICD subtypes and differences in the immune tumor microenvironment in LGG. These results may be beneficial for immunotherapy-based intervene for LGG patients, but more in-depth studies are still needed to validate our findings.

## Data availability statement

The original contributions presented in the study are included in the article/**Supplementary Material**. Further inquiries can be directed to the corresponding author.

## Ethics statement

The studies involving human participants were reviewed and approved by Ethics Committee of Xiangya Hospital in Central South University. The patients/participants provided their written informed consent to participate in this study.

## Author contributions

YK and YC conceptualized and drew up this work. BJ, YK, and YC prepared this paper. HH and XL performed analyses of the results. The images were visualized by YZ, HZ, CL, and WZ. All authors contributed to the article and approved the submitted version.
